# Malignant potential of intrahepatic biliary papillomatosis: a case report and review of the literature

**DOI:** 10.1186/1477-7819-4-71

**Published:** 2006-10-07

**Authors:** Ioannis Vassiliou, Evi Kairi-Vassilatou, Athanasios Marinis, Theodosios Theodosopoulos, Nikolaos Arkadopoulos, Vassilios Smyrniotis

**Affiliations:** 1 2^nd ^Department of Surgery, Areteion University Hospital, Athens Medical School, Athens, Greece; 2 Department of Pathology, Areteion University Hospital, Athens Medical School, Athens, Greece

## Abstract

**Background:**

Biliary papillomatosis (BP) is a rare disease entity with a strong malignant potential. It is characterized by multiple papillary adenomas involving both the intrahepatic and extrahepatic biliary tree. BP was considered in the past to be a disease with low malignant potential. However, a current review of the English literature revealed a high rate of malignant occurrence of approximately 41% and histological analysis along with the expression pattern of mucin core proteins (MUC) and mucin carbohydrate antigens suggests that BP is a borderline or low grade malignant neoplasm with a high malignant potential.

**Case presentation:**

A 68 year-old male patient was referred to our hospital due to the presence of sudden right upper quadrant abdominal pain, nausea and dark urine. Imaging workup demonstrated dilatation of the left hepatic duct without the presence of a space-occupying lesion. A left hepatectomy and cholecystectomy were carried out and histological analysis revealed a moderately to poorly differentiated carcinoma of the left hepatic duct in the background of biliary papillomatosis. Postoperative course was uneventful. Unfortunately, two years after initial diagnosis the patient rapidly deteriorated and died from multiple pulmonary secondary deposits.

**Conclusion:**

BP should not be considered to be a benign disease. The clinical behavior, the high recurrence rate and the even higher malignant transformation occurrence, as well as the presence of carcinogenetic indicators (K-ras mutation, overexpression of p53, MUC and Tn antigens) strongly support that BP is a low-grade neoplasm with high malignant potential.

## Background

Biliary papillomatosis (BP) is defined as papillary proliferation of the lining epithelium of the bile duct tree, further classified into 5 classes according to the degree of cytological and structural atypia (increased nuclear/cytoplasmic ratio, loss of polarity, hyperchromatism, pleomorphism, prominent nucleoli, abnormal mitosis, cribriform pattern and multilayering, and presence of invasion); thus, class 1 is defined as BP with low-grade atypia, class 2 as BP with high-grade atypia, class 3 as BP with in situ carcinoma, class 4 as BP with microscopic foci of stromal invasion and class 5 as BP with definite invasion into the hepatic parenchyma or fibromuscular layer of the bile duct wall [1]. In the present study a case of a class 5 BP involving the left hepatic duct is reported. A discussion of clinico-pathologic characteristics, diagnostic modalities and therapeutic management of the disease as well as a review of the English literature is presented.

## Case presentation

A 68 year-old male patient was referred to our surgical department from another hospital's medical department with a presenting clinical picture of sudden right upper quadrant abdominal pain, nausea and dark urine. With an initial diagnosis of obstructive jaundice possibly due to choledocholithiasis the patient was admitted to our department for further investigation. The patient's past medical history included upper gastrointestinal bleeding and chronic pulmonary obstructive disease.

Tumor markers (CEA, CA19-9, a-FP) were normal, while a mild elevation of the cholestatic enzymes (ALP = 138 IU/L, γ-GT = 95 IU/L) were demonstrated with return to normal of bilirubin and transaminases.

**Figure 1:**
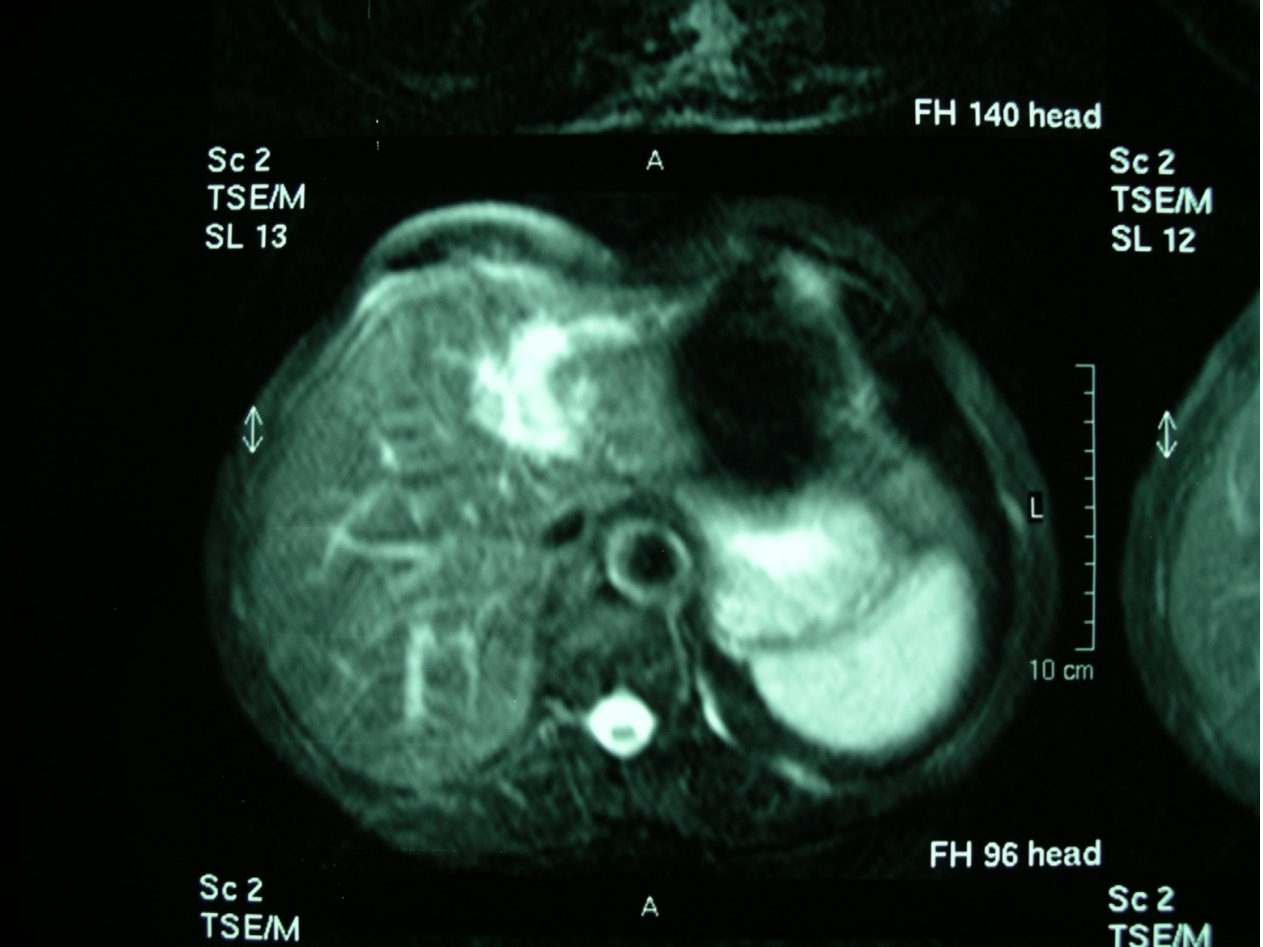
Abdominal magnetic resonance imaging demonstrating the dilated left hepatic duct, without the presence of any space-occupying lesion.

Abdominal ultrasound, computed tomography and magnetic resonance imaging (Fig. 1) demonstrated dilatation of the left intrahepatic bile ducts without the presence of any space-occupying lesion. Triplex ultrasonography of the liver confirmed the patency of portal and hepatic veins and of the hepatic artery.

Endoscopic retrograde and magnetic resonance cholangio-pancreatographies showed anomalous dilatation of the left intrahepatic bile ducts with a concomitant milder dilatation of the pancreatic duct, as well as mucus discharge from the papilla of Vater during endoscopy.

Colonoscopy was performed to rule out primary bowel neoplasm and revealed the presence of large bowel polyps. Snare polypectomies were performed and the histological analysis demonstrated the presence of tubulous and tubulovillous adenomas of the colon with mild to moderate degree of epithelial dysplasia.

Total bone scan with Tc99 m MDP, thoracic computed tomography and brain magnetic resonance imaging were negative for secondary deposits.

**Figure 2:**
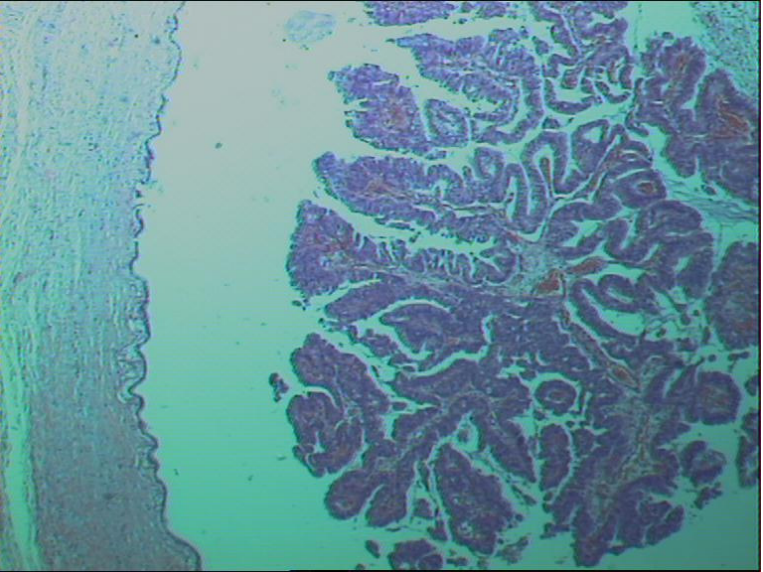
Histological section of an intrahepatic cholangiocarcinoma, papillary type (H&E stain, ×25).

**Figure 3:**
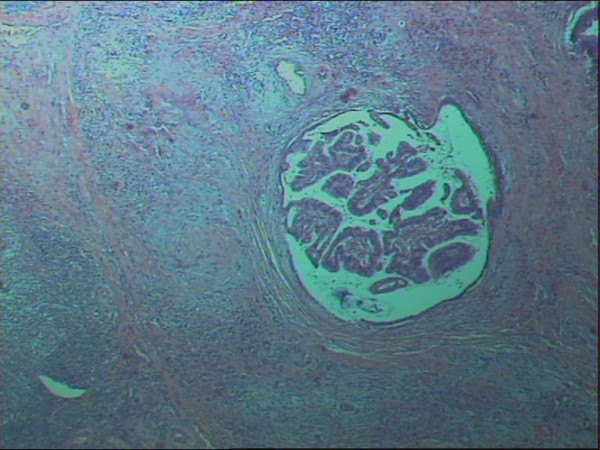
Histological section of an intraductal papillary cholangiocarcinoma showing infiltrating carcinoma of the surrounding fibrous tissue (arrow) (H&E stain, ×25).

With a diagnosis of a cholangiocarcinoma a left hepatectomy with inflow occlusion (Pringle's maneuver) and selective hepatic vascular exclusion and cholecystectomy were carried out. The histology report describes the presence of foci of papillary adenomas with a fibrovascular core connecting each of them with the ductal wall, the cuboidal or columnar cells lining the bile duct epithelium and the presence of excessive intraductal mucus, as well as foci of a moderately to poorly differentiated carcinoma, with sporadic necrotic areas and invasion of the fibrously thickened intrahepatic bile ducts (Fig 2, 3).

The postoperative course was uneventful and the patient was discharged on the 9^th ^postoperative day. Despite an uncomplicated 2 year follow-up period, the patient rapidly deteriorated and died from multiple pulmonary metastatic deposits.

## Discussion

Since the first case of biliary papillomatosis reported by Chappet in 1894 [2] approximately 140 cases have been published in the literature [1,3-5]. BP is characterized by numerous papillary tumors of variable distribution in the intrahepatic and/or extrahepatic biliary tree, extending superficially along the bile duct mucosa [1]. The course of the disease is complicated by the occurrence of malignant transformation and the consequences of chronic cholestasis due to mechanical obstruction from large amounts of mucus [5], an enlarging papilloma or tumor emboli [4], resulting in septic cholangiitis and, finally, hepatic failure [3].

Biliary papillomatosis is an unusual disease entity commonly affecting adults older than 60 years-old [4], with a male:female ratio of 2:1 [6]. In one of the greatest series reported ever, Lee et al [1] classifies BP as mucin-hypersecreting (MBP) and non-producing (NMBP), according to the presence of mucobilia which is commonly found during endoscopy. However, no differences were found in survival rates among the two groups.

The clinical picture of BP consists of recurrent colicky abdominal pain, repeated episodes of acute cholangitis with fever and jaundice, due to partial or intermittent obstruction of the bile duct by mucus, enlarging adenomas or tumor fragmentations [1,5-7]. The disease involves the extrahepatic ducts alone in 58% of cases, both extra- and intra-hepatic ducts in 33% and intrahepatic ducts alone in 9% [8].

Several pathogenetic mechanisms have been proposed, but a definite one remains to be elucidated. Recurrent pyogenic cholangitis, congenital choledochal cysts and chronic stimulation from lithiasis, infection or pancreatic juice are some of the mechanisms reported to be associated with the papillary appearance of the bile duct epithelium [4].

An enlarged intrahepatic and/or common bile duct with concomitant ill-defined filling defects consist the primary imaging features of BP. Ultrasound can demonstrate non-specific bile duct dilatation and intraductal solid masses with no distal acoustic shadowing. Endoscopic and magnetic resonance cholangiopancreatograms usually show multiple irregular filling defects. In ERCP, direct visualization of excessive mucus discharge from the papilla of Vater, as well as lack of motility on irrigation are typical endoscopic features, while the presence of intraductal masses connecting with a pedicle to the bile duct wall demonstrated in MRCP consist the key radiological features of BP [7,8]. However, diagnosis is frequently delayed due to the resemblance of the clinical picture and radiological findings to bile duct stones. Therefore, a past medical history with recurrent episodes of cholangitis and the lack of stone retrieval during ERCP should be considered as highly suspicious in differentially diagnosing BP [4]. Lai et al [9] report that the use of endoscopic ultrasonography (EUS) significantly underestimated the extent of the intrahepatic disease, emphasizing the utility of direct visualization of the biliary tree by means of choledochoscopy.

Tsui et al [10] have defined the characteristic cytologic features of BP on fine needle aspiration (FNA): a combination of hypercellular smear, very broad and often double cell layered sheets of ductal columnar epithelium, papillary configuration, preserved honeycomb pattern with even nuclear spacing and dysplastic but not frankly malignant nuclear features.

Biliary papillomatosis was considered in the past to be a disease with low malignant potential. However, a current review of the English literature revealed a high rate of malignant occurrence of approximately 41% [4]. Additionally, Lee et al [1] reports that in 83% of 58 patients with BP a coexisting carcinoma was diagnosed after taking biopsies from adenomas cholangioscopically or examining histologically the surgical specimens. Additionally, Amaya et al [11] report that histological analysis along with the expression pattern of mucin core proteins (MUC) and mucin carbohydrate antigens suggests that BP is a borderline or low grade malignant neoplasm. Furthermore, point mutations of K-ras oncogene and overexpression of p53 have been described in BP arising in a congenital choledochal cyst, without histological evidence of malignancy [12]. Furthermore, Yamashita et al [13] accomplished an immunohistological analysis in patients with hepatolithiasis and intrahepatic bile-duct carcinoma and suggest that the different expression and production of mucin carbohydrates [Tn, sialosyl-Tn(STn), and T antigens] and core proteins [MUC1-apomucin-related antigen (ARA) and MUC2-ARA] by bile-duct cystadenocarcinomas and cholangiocarcinomas are markers of a differing prognosis. Sasaki et al [14] report that intrahepatic cholangiocarcinomas extensively expressed MUC1 apomucin and focally expressed MUC2 apomucin. In addition, cholangiocarcinoma of the hilar type frequently expressed MUC3 apomucin, while MUC5/6 apomucin was more frequently expressed in well-differentiated tumors. More specifically, Shimonishi et al [15] report that epithelial hypersecretion of sialomucin rather than sulfomucin is prevalent in intraductal papillary neoplasia of the liver (IPN-L), as well is the expression of cytokeratin (CK) 20, MUC2 and nuclear p53 immunostaining. Higashi et al [16] suggest that MUC1 expression from invasive cholangiocarcinomas is associated with poor patient outcome, in contrary to expression of non-sialylated MUC2 mucin which is thought to be a favourable prognostic indicator.

Resection is the treatment of choice when BP is localized according to preoperative imaging workup and with the support of intraoperative ultrasound or cholangioscopy [4,17-19]. If the patient cannot withstand or is not willing to undergo major surgery, local ablation, stenting or drainage palliative procedures are considered [4]. In the case of diffuse BP liver transplantation is the treatment of choice [20,21]. The multicentricity and diffuse pattern of BP explains the high recurrence rate after surgical resection of the underlying lesion. Thus, bilobar or recurrent disease, as well as the high risk of malignant transformation should favor total hepatectomy and liver transplantation to be considered as the ultimate curative approach. Lee et al [3] report that after curative resection the 5-year survival rate is 81%, while in patients undergoing palliative drainage the mean survival is 37 months, significantly longer than that of cholangiocarcinoma.

## Conclusion

BP should not be considered to be a benign disease. The clinical behavior, the high recurrence rate and the even higher malignant transformation occurrence, as well as the presence of carcinogenetic indicators (K-ras mutation, overexpression of p53, MUC and Tn antigens) strongly support that BP is a low-grade neoplasm with high malignant potential. Radical surgery and liver transplantation should be considered as the only curative treatment options in order to prolong survival.

## Competing interests

The author(s) declare that they have no competing interests.

## Authors' contributions

IV and VS carried out the surgical procedures and contributed to the design of the study; AM gathered the data form the literature search, equally contributed in the preparation of the manuscript and critically revised it; EK performed the histological analysis of all surgical specimens and provided histological sections as figures for the manuscript; TT, NA and VS revised and finally approved the manuscript for been published. All authors approved the final manuscript.
